# Gene Expression by PBMC in Primary Sclerosing Cholangitis: Evidence for Dysregulation of Immune Mediated Genes

**DOI:** 10.1080/17402520600800085

**Published:** 2006

**Authors:** Christopher A. Aoki, Kevin Dawson, Thomas P. Kenny, M. Eric Gershwin, Christopher L. Bowlus

**Affiliations:** 1 Division of Gastroenterology University of California Davis PSSB #3500, 4150 V Street Sacramento CA 95817 USA; 2 Molecular and Cell Biology NIH National Center of Excellence Nutritional Genomics University of California Davis CA 95616 USA; 3 Division of Rheumatology Allergy and Clinical Immunology 451 East Health Sciences Drive, GBSF 6510 Davis CA 95616 USA

## Abstract

Primary sclerosing cholangitis (PSC) is a chronic disease of the bile ducts characterized by an inflammatory infiltrate and obliterative fibrosis. The precise role of the immune system in the pathogenesis of PSC remains unknown. We used RNA microarray analysis to identify immune-related genes and pathways that are differentially expressed in PSC. Messenger RNA (mRNA) from peripheral blood mononuclear cells (PBMC) was isolated from both patients with PSC and age and sex matched healthy controls. Samples from 5 PSC patients and 5 controls were analyzed by microarray and based upon rigorous statistical analysis of the data, relevant genes were chosen for confirmation by RT-PCR in 10 PSC patients and 10 controls. Using unsupervised hierarchical clustering, gene expression in PSC was statistically different from our control population. Interestingly, genes within the IL-2 receptor beta, IL-6 and MAP Kinase pathways were found to be differently expressed in patients with PSC compared to controls. Further, individual genes, TNF-α induced protein 6 (TNFaip6) and membrane-spanning 4-domains, subfamily A (ms4a) were found to be upregulated in PSC while similar to Mothers against decapentaplegic homolog 5 (SMAD 5) was downregulated. In conclusion, several immune-related pathways and genes were differentially expressed in PSC compared to control patients, giving further evidence that this disease is systemic and immune-mediated.


*Clinical & Developmental Immunology*, June–December 2006; 13(2–4): 265–271

Taylor & Francis

Taylor & Francis Group

### Gene expression by PBMC in primary sclerosing cholangitis: Evidence for dysregulation of immune mediated genes

CHRISTOPHER A. AOKI[1], KEVIN DAWSON[2], THOMAS P. KENNY[3], M. ERIC GERSHWIN[3], & CHRISTOPHER L. BOWLUS[1]

[1]*Division of Gastroenterology, University of California, Davis, PSSB #3500, 4150 V Street, Sacramento, CA 95817, USA,* [2]*Molecular and Cell Biology, NIH National Center of Excellence Nutritional Genomics, University of California, Davis, Davis, CA 95616, USA, and* [3]*Division of Rheumatology, Allergy and Clinical Immunology, 451 East Health Sciences Drive, GBSF 6510, Davis, CA 95616, USA*

**Abstract**

Primary sclerosing cholangitis (PSC) is a chronic disease of the bile ducts characterized by an inflammatory infiltrate and obliterative fibrosis. The precise role of the immune system in the pathogenesis of PSC remains unknown. We used RNA microarray analysis to identify immune-related genes and pathways that are differentially expressed in PSC. Messenger RNA (mRNA) from peripheral blood mononuclear cells (PBMC) was isolated from both patients with PSC and age and sex matched healthy controls. Samples from 5 PSC patients and 5 controls were analyzed by microarray and based upon rigorous statistical analysis of the data, relevant genes were chosen for confirmation by RT-PCR in 10 PSC patients and 10 controls. Using unsupervised hierarchical clustering, gene expression in PSC was statistically different from our control population. Interestingly, genes within the IL-2 receptor beta, IL-6 and MAP Kinase pathways were found to be differently expressed in patients with PSC compared to controls. Further, individual genes, TNF-α induced protein 6 (TNFaip6) and membrane-spanning 4-domains, subfamily A (ms4a) were found to be upregulated in PSC while similar to Mothers against decapentaplegic homolog 5 (SMAD 5) was downregulated. In conclusion, several immune-related pathways and genes were differentially expressed in PSC compared to control patients, giving further evidence that this disease is systemic and immune-mediated.

**Keywords:** *Microarray, TNF-α induced protein 6 (TNFaip6), membrane-spanning 4-domains subfamily A (ms4a), similar to mothers against decapentaplegic homolog 5 (SMAD 5)*

### Introduction

Primary sclerosing cholangitis (PSC) is a rare liver disease of chronic inflammation affecting primarily medium and large sized bile ducts leading to fibrosis and eventually cirrhosis. Histologically, PSC is characterized by an inflammatory infiltrate and obliterative fibrosis, which leads to the pathognomic biliary strictures observed on cholangiographic imaging. Whether this immune response is autoimmune in nature or simply a response to bacterial or viral pathogens remains unresolved.

Several lines of evidence have established that PSC is an immune-mediated process. First, the inflammatory infiltrate of PSC consists predominantly of T cells, which have been shown to have a Th-1 type phenotype. Second, patients with PSC and their family members have increased frequencies of autoimmune diseases (Saarinen et al. 2000; Bergquist et al. 2005), for example, approximately 80% of patients with PSC have coexistent inflammatory bowel disease (IBD), most often ulcerative colitis (Schrumpf and Boberg 2001; Bambha et al. 2003). Third, the human leukocyte antigen (HLA) complex, particularly the HLA B8-DR3 haplotype, has been positively associated with PSC (Schrumpf et al. 1982; Chapman et al. 1983; Spurkland et al. 1999; Donaldson and Norris 2001; Wiencke et al. 2001; Bittencourt et al. 2002; Neri et al. 2003). Finally, autoantibodies are frequent in PSC, the most

Correspondence: C. L. Bowlus, Division of Gastroenterology, University of California, Davis, 4150 V Street, PSSB 3500, Sacramento, CA 95817, USA. Tel: 1 916 734 7183. Fax: 1 916 734 7908. E-mail: clbowlus@ucdavis.edu

ISSN 1740-2522 print/ISSN 1740-2530 online © 2006 Taylor & Francis
DOI: 10.1080/17402520600800085

common is the atypical perinuclear anti-neutrophil antibody (p-ANCA), present in 80% of patients (Terjung and Worman 2001; Aoki et al. 2005b). The purpose of this study was to compare the gene expression profile of peripheral blood mononuclear cells (PBMC) from PSC patients with age and sex matched controls using microarray technology.

### Materials and methods

### *Subjects*

The PSC patients were clinically well characterized and diagnosed in accordance with accepted criteria with typical findings at cholangiography (Chapman et al. 1980; Wiesner and LaRusso 1980), the diagnosis and classification of IBD were based on endoscopic and histological criteria. Demographic and clinical characteristics of the patients are noted in Table I. Unrelated, healthy, age and sex matched controls were used throughout this study. The study protocol conformed to the ethical guidelines of the 1975 Declaration of Helsinki as reflected in *a priori* approval by the appropriate institutional review committee. Informed consent in writing was obtained from each subject.

### *RNA isolation*

Heparinized venous blood samples were obtained and within 1 h of collection, PBMC isolated by Ficoll-Histopaque gradient centrifugation techniques using Histopaque-1077 (Sigma Chemical Co, St Louis, MO, USA). Trizol Reagent (Invitrogen, Carlsbad, CA, USA) was used to isolate total RNA, the RNA quality and integrity was determined using an Agilent 2100 Bioanalyzer (Agilent Technologies, Palo Alto, CA, USA) and absorbance at A260/A280.

### *Microarray analysis*

In the first part of this study, five PSC subjects (PSC 1, 3, 4, 5 and 7) and matched controls were used for microarray analysis. Sample labeling, hybridization to chips and image scanning were performed as described (Affymetrix Expression Analysis Technical Manual). For each sample, 10 μg total RNA was labeled using One-Cycle Target Labeling and hybridized to the Affymetrix Human U133 Plus2 arrays that contain more that 54,000 probe sets that sample over 39,000 genes.

### *Data analysis*

The ten CEL files were first imported into the R environment and analysed with the Affymetrix® package of Bioconductor. Raw data were normalized with quantile normalization; background corrected and gene expression levels computed with Robust Multichip Average (RMA) (http://www.bioconductor.org/). Presence/absence calls were computed using the Wilcoxon signed rank-based gene expression presence/absence detection algorithm. Genes present with a $p < 0.005$ significance level were included for further analysis. Significant differences between PSC and control groups were computed using Student $t$-test for genes that differed at least 2-fold between the medians of the cases and controls. A $p$-value of $p < 0.005$ was considered significant. Genes that were expressed in at least 80% of the samples and had a 2-fold or greater difference compared to the median of the samples were clustered using unsupervised hierarchical clustering with Gene Cluster 3.0. The leaves of the clustering trees were then folded with Self-Organizing Map (SOM) algorithm. The clustering result was presented using Java TreeView 1.0.1.

### *Semi-quantitative RT-PCR*

For the second part of our study, mRNA from 10 PSC patients and 10 aged and sex matched controls were used for RT-PCR. Gene microarray results were validated for a select number of transcripts using semi-quantitative real-time polymerase chain reaction (Table II) performed on 1 μl of a 1:10 dilution of the cDNA reaction was used for template with primers in a 25-μl SYBR Green Assay

Table I. Characteristics of patients with PSC.

| PSC Patients | Age (y) | Sex | Co-morbidities | Relevant medications | Disease duration (years) | Disease stage |
|---|---|---|---|---|---|---|
| 1 | 56 | Female | UC | UDCA/mesalamine | 5 | Unknown |
| 2 | 55 | Female | UC | UDCA | 10 | Grade I |
| 3 | 26 | Female | UC | Vancomycin | 2 | Grade II |
| 4 | 60 | Male | UC | Folic acid | 10 | Cirrhosis |
| 5 | 32 | Male | None | UDCA | 12 | Cirrhosis |
| 6 | 39 | Female | None | UDCA | 4 | Grade III |
| 7 | 58 | Male | CD | Mesalamine | 1 | None |
| 8 | 33 | Female | CD, Sacroilitis | None | 10 | Cirrhosis |
| 9 | 56 | Male | None | None | 4 | Cirrhosis |
| 10 | 46 | Male | None | UDCA | 13 | Cirrhosis |

UC, ulcerative colitis; CD, Crohn's disease; UDCA, ursodeoxycholic acid.

Table II. Genes chosen for confirmation by RT-PCR and their primer sequences.

| Genes | Affymetrix probe ID | Forward primer sequence | Reverse primer sequence |
|---|---|---|---|
| NFKB | N.A. | GGCAGCAAATAGACGAGCTCC | CGCCGCTGTCGCAGAC |
| Sara1a | 210790_s_at | TTCCAACACTACATCCGACATCA | AGGTCATTCCAGCAATTGTTAGCT |
| Smad 5 | 205187_at | CAGCAAGTTCTGGACCAGGAA | GGAGGCGTATCAGCTGGGA |
| Smad 4 | 1565703_at | CCGGACATTACTGGCCTGTT | AAATGGGAGGCTGGAATGC |
| TGFBRI | 206943_at | CCTGGCTAGAGAAGCACAAAAAGTAT | AATGATAGAGCAGTCCAAGTGCAA |
| TNFaip6 | 206026_s_at | ACAACCCACACGCAAAGGAG | TTTGGGAAGCCTGGAGATTTAA |
| Msa4a | 1555728_a_at | GGATCCTTGTCAATTGCAGCA | TCGGACCAGGCCTTTTGTAGT |
| Hmtp1 | 216069_at | TGAGGTAAGCATTGCCCTAA | ATTGAACCAGCCTTGCATTC |
| IREBP2 | 1563130_a_at | GCATAACCTTTGGCACTTCAA | TCATCTTTTGAAGCCAAGCA |

(PE Biosystems, Foster City, CA, USA) reaction. Cycling consisted of one-cycle at 50°C for 2 min, one-cycle at 95°C for 2 min, and 40 cycles of 95°C for 15 s and 60°C for 1 min on an ABI 7700 Sequence Detector (PE Biosystems, Foster City, CA, USA). GAPDH RNA was also measured using primers GAPDH.F (5′-GAAGGTGAAGGTCGGAGTC-3′) and GAPDH.R (5′-GAAGATGGTGATGGGA-TTTC-3′). All samples were assayed in duplicate. Quantification was performed using Sequence

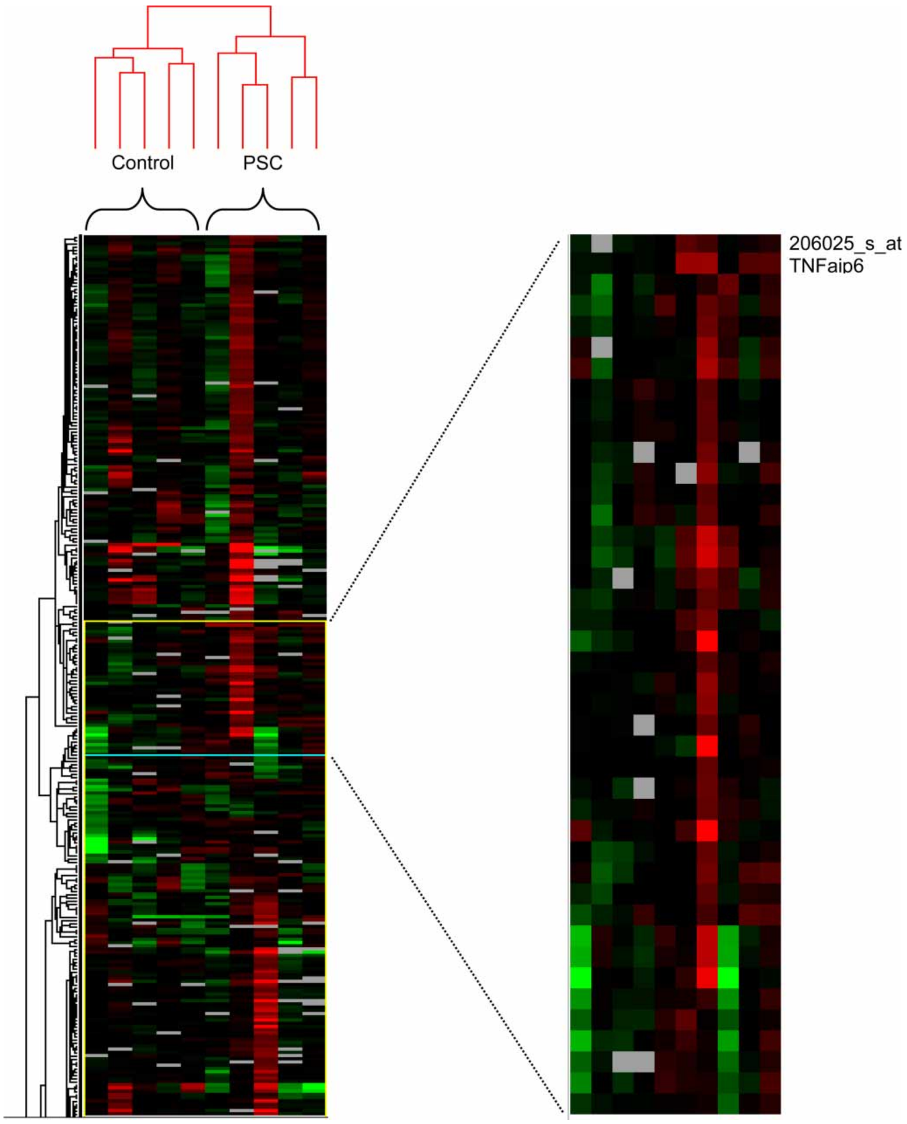

Figure 1. Unsupervised hierarchical clustering differentiated the gene expression arrays from 5 PSC patients and 5 age- and sex-matched controls. Two oligo sets for the TNFAIP6 gene are shown. Gene expressions with higher than median values are shown in red, those with lower than median values are shown in green. Gene expression values found missing are labeled gray.

Table III. Potential pathways that may be disrupted in patients with PSC based on the gene expression profiles of PBMC.

| Pathway/gene (accession) | Fold-change | *p*-value |
|---|---|---|
| *IL-2 receptor beta activation of T cells* | | |
| JAK3 | 1.6 | 0.002 |
| SOCS3 | 1.4 | 0.001 |
| BCL2 | 1.2 | 0.002 |
| PPIA | 0.8 | 0.004 |
| CFLAR | 0.6 | 0.001 |
| *IL-6 signaling* | | |
| JAK3 | 1.6 | 0.002 |
| STAT3 | 1.2 | 0.002 |
| SHP2 | 0.8 | 0.001 |
| IL6RB | 0.7 | 0.002 |

Table IV. Genes with the highest fold difference in expression in PSC compared to controls when analyzed by *t*-test and fold change compared to median of the control samples.

| Gene and affymetrix ID | Fold | *p*-value |
|---|---|---|
| 219607_s_at \| membrane-spanning 4-domains, subfamily A, member 4 | 3.33 | 0.005 |
| 202887_s_at \| DNA-damage-inducible transcript 4 | 2.83 | 0.002 |
| 202912_at \| adrenomedullin | 2.45 | 0.0004 |
| 206026_s_at \| tumor necrosis factor, alpha-induced protein 6 | 2.37 | 0.005 |
| 228325_at \| KIAA0146 protein | 2.35 | 0.001 |

Detector version 1.7 software. A standard curve was established with pooled cDNA derived from the age and sex matched controls and used as a calibrator in each assay. mRNA was recorded relative to the standard and normalized to GAPDH. Statistical analysis was performed using Graphpad Prism v 4.0 (Graphpad Inc, San Diego, CA, USA). Because the distribution was non-Gaussian, comparisons were made with non-parametric methods. A *P* value of less than 0.05 was considered significant.

### Results

Unsupervised hierarchical clustering separated PSC cases from controls (Figure 1). 1887 genes were clustered that were expressed in at least 80% of the samples with at least one of the samples having a 2-fold difference in expression compared to the median of the samples. Using a *t*-test comparison we identified 942 genes differentially expressed in patients with PSC compared to controls when significance was set at $p < 0.005$. Using the Database for Annotation and Visual Integration Discovery (DAVID) on these 942 genes we found evidence of several pathways potentially altered in PSC (Table III). These pathways included IL-2 receptor beta activation of T cells, IL-6 signaling, macrophage differentiation by M-CSF and MAP kinase-signaling pathways. We discovered in PSC PBMC three genes expressed greater than 2-fold and 18 genes expressed less than 0.5-fold the median expression index of the controls (Tables III, IV and V).

To validate the microarray data and to investigate potential pathways suggested by the array data, we chose nine genes to quantify by RT-PCR in10 PSC cases and 10 controls (Figure 2). Of the genes with greater than 2-fold expression in PSC by microarray, membrane-spanning 4-domains subfamily A member 4 and tumor necrosis factor alpha-induced protein six were found to be expressed significantly greater in PSC by RT-PCR. SMAD 5, which was 0.6-fold less than controls by microarray at a *P* value of 0.00003, was found to be significantly lower in PSC than controls. However, SMAD 4 and TGF-β receptor I which signals through SMAD 4 and SMAD 5 were not found to be significantly different between PSC and controls. No other genes tested were found to have statistically significant differences between PSC patients and controls by RT-PCR (data not shown).

### Discussion

Using microarray technology, we screened PBMC from PSC patients in search of genes that might lead to insights in the pathophysiology of PSC. We chose to analyse this data with maximum sensitivity using a simple *t*-test and fold change comparison to the median of the control sample. From this data we then sought to determine immunological pathways that may be relevant to the disease. Analysis of the results suggested the involvement of the IL-2 receptor beta activation of T cells and IL-6 signaling. The IL-2 receptor is composed of the alpha, beta and gamma subunit (Leonard et al. 1984). In addition to resting and activated T cells, the IL-2 receptor beta has been found on B cells (Zola et al. 1991), natural killer cells (Nishikawa et al. 1990), monocytes (Espinoza-Delgado et al. 1990), dendritic epidermal cells (Tanaka et al. 1992) and neutrophils (Djeu et al. 1993). IL-2 receptor beta knock out mice develop an increase in activated $CD4^{+}$T cells, an increase in total IgG, high-titers of anti-nuclear and anti-DNA autoantibodies, and an autoimmune hemolytic anemia. Interestingly, these mice also have an autoimmune mediated liver disease with immature myeloid cell infiltration of the liver sinusoids and portal tracts (Suzuki et al. 1995). The differentially expressed genes in this pathway found in patients with PSC may reflect a downregulation of the IL-2 receptor beta and potentially plays a role in the development of inflammation of the medium and large bile ducts observed in PSC.

The reduced expression of SMAD 5 in PSC may be relevant as a number of autoimmune diseases

Table V. Genes with the lowest fold difference in PSC patients when compared to controls when analyzed by *t*-test and fold change compared to the median of the control samples.

| Gene and affymetrix ID | Fold | *p*-value |
|---|---|---|
| 209728_at \| major histocompatibility complex, class II, DR beta 4 | 0.026 | 0.00000003 |
| 215666_at \| major histocompatibility complex, class II, DR beta 4 | 0.101 | 0.0004 |
| 243037_at \| far upstream element (FUSE) binding protein 1 | 0.398 | 0.0007 |
| 1563130_a_at \| iron-responsive element binding protein 2 | 0.439 | 0.00006 |
| 235547_at \| phosphonoformate immuno-associated protein 5 | 0.444 | 0.0009 |
| 216069_at \| HMT1 hnRNP methyltransferase-like 1 (S. cerevisiae) | 0.455 | 0.00005 |
| 233940_at \| echinoderm microtubule associated protein like 4 | 0.463 | 0.004 |
| 243751_at \| NA | 0.464 | 0.003 |
| 229413_s_at \| ring finger protein 3 | 0.471 | 0.002 |
| 215586_at \| protein phosphatase 3 (formerly 2B), catalytic subunit, beta isoform (calcineurin A beta) | 0.473 | 0.0008 |
| 242894_at \| NA | 0.477 | 0.002 |
| 236353_at \| NA | 0.478 | 0.004 |
| 230742_at \| RNA binding motif protein 5 | 0.479 | 0.002 |
| 226085_at \| chromobox homolog 5 (HP1 alpha homolog, Drosophila) | 0.481 | 0.004 |
| 242144_at \| PAP associated domain containing 4 | 0.485 | 0.002 |
| 239258_at \| phosphatidylinositol glycan, class F | 0.489 | 0.004 |
| 1557270_at \| NA | 0.491 | 0.001 |
| 244219_at \| Wilms tumor 1 associated protein | 0.494 | 0.00003 |

NA, transcript with no known gene.

including IBD have been reproduced in TGFβ knock out mice (Hahm et al. 2001; Aoki et al. 2005a). TGFβ has immunosuppressive properties that are mediated by SMAD signaling.

Other genes confirmed to be differentially expressed in PSC compared to control include TNF-α induced protein 6 (TNFaip6) and membrane-spanning 4-domains, subfamily A (ms4a). TNFaip6 is a 35 kilodalton (kda) protein that is elevated in a number of autoimmune diseases including, systemic lupus erythematosus, rheumatoid arthritis (Wisniewski and Vilcek 1997) and mucosal smooth muscle cells in IBD (Wisniewski and Vilcek 1997). TNFaip6 is a secreted protein (Lee et al. 1992) found also on PBMC after stimulation with LPS, TNFα, PHA and Con A, but is not present on PBMC from healthy human subjects (Lee et al. 1993; Wisniewski et al. 1993). TNFaip6 is believed to contribute to chronic inflammation in a

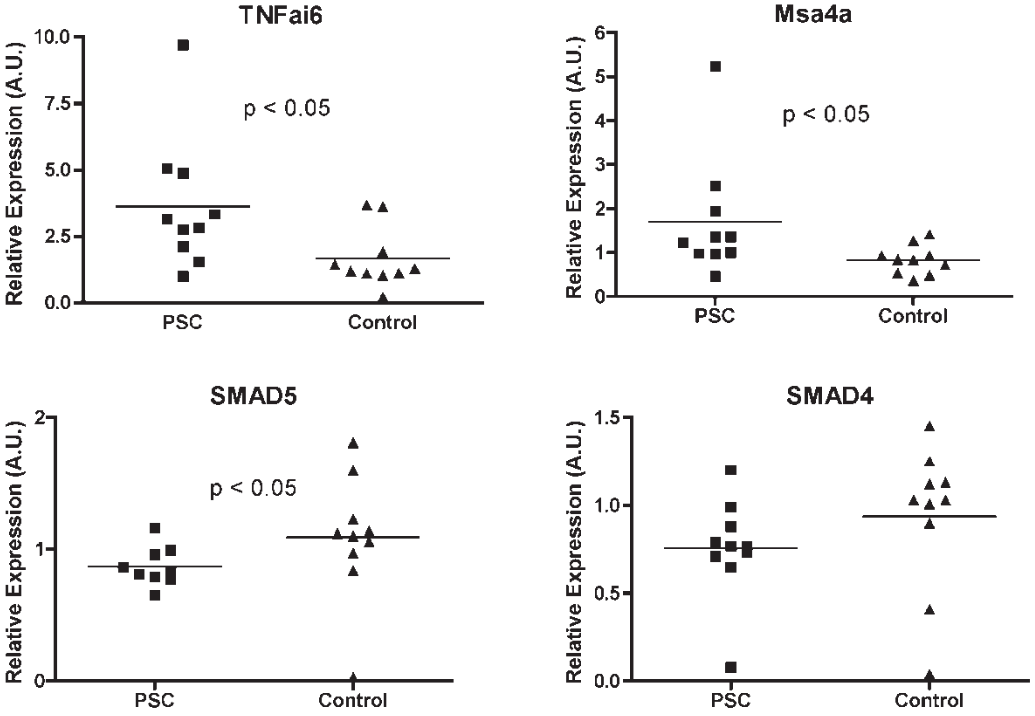

Figure 2. Confirmatory RT-PCR using cDNA derived from PBMC from patients with PSC compared to age- and sex-matched healthy control patients. Each gene is normalized to GAPDH and relative to pooled cDNA from the controls. The horizontal line represents the mean expression.

number of ways. First, secreted TNFaip6 complexes with a serine protease inhibitor, inter-alpha-inhibitor (Wisniewski et al. 1994), these complexes have been found in the synovial fluid of rheumatoid arthritis patients and tightly bind hyaluronic acid (HA), a major component of extra cellular matrix tissue. The formation of the TNFaip6:inter-alpha-inhibitor may play a role in disease by binding to HA and cause a dysregulation of extracellular matrix remodeling or assembly (Milner and Day 2003). This may be occurring in the extracellular matrix of PSC patients where there is a chronic fibrosing process surrounding biliary epithelial cells, resulting in an "onion skin" like appearance on histopathology. Alternatively, the upregulation of TNFaip6 on PBMC in patients with PSC may be a marker of intense TNFα or IL-1 stimulation and is acting to inhibit the inflammatory process as has been shown in air pouch mice, a mouse model of arthritis (Wisniewski et al. 1996).

MSa4a is a member of a superfamily of which includes CD20, a marker of precursor and mature B cells, high affinity IgE receptor (FcεR1β) found on mast cells and basophils, and a hematopoietic-cell specific protein found on a diverse group of myeloid and lymphoid cells (HTm4) of unknown function (Ishibashi et al. 2001). These proteins share 16–32% homology and their genes are all found on chromosome 11q12–13.1 (Tedder et al. 1988; Hupp et al. 1989; Adra et al. 1994). Interestingly, this gene has been mapped to a susceptibility locus in patients with asthma/atopy (Adra et al. 1999). The MSa4a is more closely related to Htm4 and FcεR1β than CD 20 and found on a number of human lymphoblastoid cell lines, but more highly expressed in pre B cell (NALM-6) and B cell lines (BJAB) than T cell lines (Liang and Tedder 2001). This may confirm prior reports, which have shown B cells to be the predominant cell type in PBMC in patients with PSC (Lindor et al. 1987). The function of the Msa4a protein is unknown but given its amino acid homology to the other members of the superfamily it is suggested it is a component of an oligomeric cell surface complex that plays a role in signal transduction (Liang and Tedder 2001).

A number of other candidate genes remain to be examined further using RT-PCR. We have initially chosen only those thought to be immunologically relevant; for many of the genes differentially expressed, the function remains to be identified. In addition, further work needs to be performed, to define the cell type and protein expression of the identified genes. There has been one other study looking at genome wide expression in autoimmune liver disease (Shackel et al. 2001). RNA was extracted from liver biopsies of patients with PBC ($n = 6$), PSC ($n = 4$) and normal liver and the cDNA array used consisted of 588 genes and nine housekeeping genes. None of the genes differentially expressed in that study were identified in this present study. This lack of correlation may be related to the different tissue and gene chip used in that prior study. Our demonstration of several immune-related pathways and genes differentially expressed in PSC PBMC compared to control patients, suggests that the disease is more than a local inflammatory process in the liver and reflects a systemic immune response.

### References

Adra CN, Lelias JM, Kobayashi H, Kaghad M, Morrison P, Rowley JD, Lim B. 1994. Cloning of the cDNA for a hematopoietic cell-specific protein related to CD20 and the beta subunit of the high-affinity IgE receptor: Evidence for a family of proteins with four membrane-spanning regions. Proc Natl Acad Sci USA 91(21):10178–10182.

Adra CN, Mao XQ, Kawada H, Gao PS, Korzycka B, Donate JL, Shaldon SR, Coull P, Dubowitz M, Enomoto T, et al. 1999. Chromosome 11q13 and atopic asthma. Clin Genet 55(6):431–437.

Aoki CA, Borchers AT, Li M, Flavell RA, Bowlus CL, Ansari AA, Gershwin ME. 2005a. Transforming growth factor beta (TGF-beta) and autoimmunity. Autoimmun Rev 4(7):450–459.

Aoki CA, Bowlus CL, Gershwin ME. 2005b. The immunobiology of primary sclerosing cholangitis. Autoimmun Rev 4(3):137–143.

Bambha K, Kim WR, Talwalkar J, Torgerson H, Benson JT, Therneau TM, Loftus EV, Jr, Yawn BP, Dickson ER, Melton LJ, 3rd. 2003. Incidence, clinical spectrum, and outcomes of primary sclerosing cholangitis in a United States community. Gastroenterology 125(5):1364–1369.

Bergquist A, Lindberg G, Saarinen S, Broome U. 2005. Increased prevalence of primary sclerosing cholangitis among first-degree relatives. J Hepatol 42(2):252–256.

Bittencourt PL, Palacios SA, Cancado EL, Carrilho FJ, Porta G, Kalil J, Goldberg AC. 2002. Susceptibility to primary sclerosing cholangitis in Brazil is associated with HLA-DRB1*13 but not with tumour necrosis factor alpha -308 promoter polymorphism. Gut 51(4):609–610.

Chapman RW, Arborgh BA, Rhodes JM, Summerfield JA, Dick R, Scheuer PJ, Sherlock S. 1980. Primary sclerosing cholangitis: A review of its clinical features, cholangiography, and hepatic histology. Gut 21(10):870–877.

Chapman RW, Varghese Z, Gaul R, Patel G, Kokinon N, Sherlock S. 1983. Association of primary sclerosing cholangitis with HLA-B8. Gut 24(1):38–41.

Djeu JY, Liu JH, Wei S, Rui H, Pearson CA, Leonard WJ, Blanchard DK. 1993. Function associated with IL-2 receptor-beta on human neutrophils. Mechanism of activation of antifungal activity against Candida albicans by IL-2. J Immunol 150(3):960–970.

Donaldson PT, Norris S. 2001. Immunogenetics in PSC. Best Pract Res Clin Gastroenterol 15(4):611–627.

Espinoza-Delgado I, Ortaldo JR, Winkler-Pickett R, Sugamura K, Varesio L, Longo DL. 1990. Expression and role of p75 interleukin 2 receptor on human monocytes. J Exp Med 171(5):1821–1826.

Hahm KB, Im YH, Parks TW, Park SH, Markowitz S, Jung HY, Green J, Kim SJ. 2001. Loss of transforming growth factor beta signalling in the intestine contributes to tissue injury in inflammatory bowel disease. Gut 49(2):190–198.

Hupp K, Siwarski D, Mock BA, Kinet JP. 1989. Gene mapping of the three subunits of the high affinity FcR for IgE to mouse chromosomes 1 and 19. J Immunol 143(11):3787–3791.

Ishibashi K, Suzuki M, Sasaki S, Imai M. 2001. Identification of a new multigene four-transmembrane family (MS4A) related to

CD20, HTm4 and beta subunit of the high-affinity IgE receptor. Gene 264(1):87–93.

Lee TH, Wisniewski HG, Vilcek J. 1992. A novel secretory tumor necrosis factor-inducible protein (TSG-6) is a member of the family of hyaluronate binding proteins, closely related to the adhesion receptor CD44. J Cell Biol 116(2):545–557.

Lee TH, Klampfer L, Shows TB, Vilcek J. 1993. Transcriptional regulation of TSG6, a tumor necrosis factor- and interleukin-1-inducible primary response gene coding for a secreted hyaluronan-binding protein. J Biol Chem 268(9):6154–6160.

Leonard WJ, Depper JM, Crabtree GR, Rudikoff S, Pumphrey J, Robb RJ, Kronke M, Svetlik PB, Peffer NJ, Waldmann TA, et al. 1984. Molecular cloning and expression of cDNAs for the human interleukin-2 receptor. Nature 311(5987):626–631.

Liang Y, Tedder TF. 2001. Identification of a CD20-, FcepsilonRI-beta-, and HTm4-related gene family: Sixteen new MS4A family members expressed in human and mouse. Genomics 72(2):119–127.

Lindor KD, Wiesner RH, Katzmann JA, LaRusso NF, Beaver SJ. 1987. Lymphocyte subsets in primary sclerosing cholangitis. Dig Dis Sci 32(7):720–725.

Milner CM, Day AJ. 2003. TSG-6: A multifunctional protein associated with inflammation. J Cell Sci 116(Pt 10): 1863–1873.

Neri TM, Cavestro GM, Seghini P, Zanelli PF, Zanetti A, Savi M, Podda M, Zuin M, Colombo M. Floreani A, et al. 2003. Novel association of HLA-haplotypes with primary sclerosing cholangitis (PSC) in a southern European population. Dig Liver Dis 35(8):571–576.

Nishikawa K, Saito S, Morii T, Kato Y, Narita N, Ichijo M, Ohashi Y, Takeshita T, Sugamura K. 1990. Differential expression of the interleukin 2 receptor beta (p75) chain on human peripheral blood natural killer subsets. Int Immunol 2(6):481–486.

Saarinen S, Olerup O, Broome U. 2000. Increased frequency of autoimmune diseases in patients with primary sclerosing cholangitis. Am J Gastroenterol 95(11):3195–3199.

Schrumpf E, Fausa O, Forre O, Dobloug JH, Ritland S, Thorsby E. 1982. HLA antigens and immunoregulatory T cells in ulcerative colitis associated with hepatobiliary disease. Scand J Gastroenterol 17(2):187–191.

Schrumpf E, Boberg KM. 2001. Epidemiology of primary sclerosing cholangitis. Best Pract Res Clin Gastroenterol 15(4):553–562.

Shackel NA, McGuinness PH, Abbott CA, Gorrell MD, McCaughan GW. 2001. Identification of novel molecules and pathogenic pathways in primary biliary cirrhosis: cDNA array analysis of intrahepatic differential gene expression. Gut 49(4):565–576.

Spurkland A, Saarinen S, Boberg KM, Mitchell S, Broome U, Caballeria L, Ciusani E, Chapman R, Ercilla G, Fausa O, et al. 1999. HLA class II haplotypes in primary sclerosing cholangitis patients from five European populations. Tissue Antigens 53(5):459–469.

Suzuki H, Kundig TM, Furlonger C, Wakeham A, Timms E, Matsuyama T, Schmits R, Simard JJ, Ohashi PS, Griesser H, et al. 1995. Deregulated T cell activation and autoimmunity in mice lacking interleukin-2 receptor beta. Science 268(5216):1472–1476.

Tanaka T, Takeuchi Y, Shiohara T, Kitamura F, Nagasaka Y, Hamamura K, Yagita H, Miyasaka M. 1992. In utero treatment with monoclonal antibody to IL-2 receptor beta-chain completely abrogates development of Thy-1 + dendritic epidermal cells. Int Immunol 4(4):487–491.

Tedder TF, Klejman G, Disteche CM, Adler DA, Schlossman SF, Saito H. 1988. Cloning of a complementary DNA encoding a new mouse B lymphocyte differentiation antigen, homologous to the human B1 (CD20) antigen, and localization of the gene to chromosome 19. J Immunol 141(12):4388–4394.

Terjung B, Worman HJ. 2001. Anti-neutrophil antibodies in primary sclerosing cholangitis. Best Pract Res Clin Gastroenterol 15(4):629–642.

Wiencke K, Spurkland A, Schrumpf E, Boberg KM. 2001. Primary sclerosing cholangitis is associated to an extended B8-DR3 haplotype including particular MICA and MICB alleles. Hepatology 34(4 Pt 1):625–630.

Wiesner RH, LaRusso NF. 1980. Clinicopathologic features of the syndrome of primary sclerosing cholangitis. Gastroenterology 79(2):200–206.

Wisniewski HG, Maier R, Lotz M, Lee S, Klampfer L, Lee TH, Vilcek J. 1993. TSG-6: A TNF-, IL-1-, and LPS-inducible secreted glycoprotein associated with arthritis. J Immunol 151(11):6593–6601.

Wisniewski HG, Burgess WH, Oppenheim JD, Vilcek J. 1994. TSG-6, an arthritis-associated hyaluronan binding protein, forms a stable complex with the serum protein inter-alpha-inhibitor. Biochemistry 33(23):7423–7429.

Wisniewski HG, Hua JC, Poppers DM, Naime D, Vilcek J, Cronstein BN. 1996. TNF/IL-1-inducible protein TSG-6 potentiates plasmin inhibition by inter-alpha-inhibitor and exerts a strong anti-inflammatory effect *in vivo*. J Immunol 156(4):1609–1615.

Wisniewski HG, Vilcek J. 1997. TSG-6: An IL-1/TNF-inducible protein with anti-inflammatory activity. Cytokine Growth Factor Rev 8(2):143–156.

Zola H, Weedon H, Thompson GR, Fung MC, Ingley E, Hapel AJ. 1991. Expression of IL-2 receptor p55 and p75 chains by human B lymphocytes: Effects of activation and differentiation. Immunology 72(2):167–173.

